# The social determinants of otitis media in aboriginal children in Australia: are we addressing the primary causes? A systematic content review

**DOI:** 10.1186/s12889-020-08570-3

**Published:** 2020-04-15

**Authors:** Jack DeLacy, Tinashe Dune, John J. Macdonald

**Affiliations:** 1 Children’s Hospital Westmead Clinical School, The University of Sydney & The Sax Institute (Study of Environment on Aboriginal Resilience and Child Health), Glebe, Australia; 2 School of Health Sciences, Western Sydney University, Sydney, Australia; 3 Translational Health Research Institute, Western Sydney University, Sydney, Australia; 4 Men’s Health Information and Resources Centre, Western Sydney University, Sydney, Australia

**Keywords:** Otitis media, Ear disease, Hearing, Social determinants of health, Aboriginal and Torres Strait islander, Aboriginal, Indigenous, Children, Australia

## Abstract

**Background:**

Aboriginal and Torres Strait Islander children experience some of the highest rates of otitis media in the world. Key risk factors for otitis media in Aboriginal children in Australia are largely social and environmental factors such as overcrowded housing, poverty and limited access to services. Despite this, little is known about how to address these risk factors. A scoping content review was performed to determine the relationship between social determinants of health and otitis media in Aboriginal and Torres Strait Islander children as described by peer-reviewed and grey literature.

**Method:**

Search terms were established for location, population and health condition. The search terms were used to conduct a literature search using six health research databases. Following the exclusion process, articles were scoped, analysed and categorised using scoping parameters and a social determinants of health framework.

**Results:**

Housing-related issues were the most frequently reported determinants for otitis media (56%). Two articles (4%) directly investigated the impact of social determinants of health on rates of otitis media within Aboriginal and Torres Strait Islander children. The majority of the literature (68%) highlights social determinants as playing a key role in the high rates of otitis media seen in Aboriginal populations in Australia. There were no intervention studies targeting social determinants as a means to reduce otitis media rates among Aboriginal and Torres Strait Islander children.

**Conclusions:**

This review identifies a disconnect between otitis media drivers and the focus of public health interventions within Aboriginal and Torres Strait Islander populations. Despite consensus that social determinants play a key role in the high rates of otitis media in Aboriginal and Torres Strait Islander children, the majority of intervention studies within the literature are focussed on biomedical approaches such as research on vaccines and antibiotics. This review highlights the need for otitis media intervention studies to shift away from a purely biomedical model and toward investigating the underlying social determinants of health. By shifting interventions upstream, otitis media rates may decrease within Aboriginal and Torres Strait Islander children, as focus is shifted away from a treatment-focussed model and toward a more preventative model.

## Background

Otitis media (OM) is one of the leading causes of disease among Aboriginal and Torres Strait Islander (hereafter referred to as Aboriginal) children [1, 2]. OM refers to inflammation and infection of the middle ear and is classified as acute OM, OM with effusion or chronic suppurative OM [1, 3]. There are currently inadequate services to deal with ear and hearing health within Aboriginal communities and high demand for services is expected to continue in coming years [4]. The World Health Organisation have identified OM in its various forms as a major health issue for Aboriginal children, despite the fact that OM is preventable and treatable, and is far less common for non-Aboriginal children in Australia [1]. The gap in prevalence of OM between Aboriginal and non-Aboriginal children has consistently been associated with social determinants, particularly housing-related issues [2–8]. OM can impact upon educational outcomes and employability for Aboriginal people who are more likely to be socially and economically disadvantaged than non-Aboriginal Australians [5].

Key risk factors for OM in Aboriginal children include overcrowded housing, poor housing conditions, exposure to tobacco smoke, malnutrition, socioeconomic disadvantage and limited access to services [2–8]. Aboriginal children experience OM at similar rates, frequency and severity as children living in developing nations, despite the overall high standard of living in Australia [9, 10]. The prevalence of OM in some Aboriginal communities is close to 10 times higher than the 4% identified by The World Health Organisation as being a serious public health problem requiring urgent attention [2]. This puts Aboriginal children as one of the most at risk populations for OM in the world [3, 11].

Significant health gaps have persisted in Aboriginal populations since the British invaded Australia in 1788 [6, 10]. These health gaps are highlighted by the gap in life-expectancy between Aboriginal and non-Aboriginal Australians, with Aboriginal children born between 2010 and 2012 expected to live 10.05 years younger than non-Aboriginal children [12]. Furthermore, social and economic disadvantage have been underscored as significant contributing factors to these poor health outcomes [7]. Therefore, social determinants of OM in Aboriginal children need to be better understood in light of evidence supporting the impact of poor housing, exposure to tobacco smoke and socioeconomic disadvantage on the prevalence and persistence of OM in Aboriginal children.

This review aims to identify how social determinants are addressed in grey and peer-reviewed literature, regarding drivers of OM and proposed interventions aimed at minimising the health burden of OM among Aboriginal children. This review aims to identify gaps in the literature and guide further research, policy development and service provision.

## Methods

Given the broad nature of the research objective, a scoping content review was conducted to explore available research, to evaluate the need for further investigation, to describe key themes and to identify gaps in the literature. The framework proposed by Arksey and O’Malley [13] for conducting a scoping content review was adapted for this study and is detailed below.

### Research question and search strategy

Initially, the research objective was established through preliminary review of the literature and discussion between the research team. Following the establishment of the research objective, the search strategy was developed by implementing inclusion and exclusion criteria, and keywords (see Table 1). The location was limited to Australia, the included literature was limited to English only and no time constraints were placed on the date of publication. The population of focus was established by two criteria: individuals of Australian Aboriginal identity and children aged 12 years old or younger. Health condition terms related to OM and ear disease. Literature type included peer-reviewed and grey literature.

**Table 1:** Search Strategy and Keywords

Parameters	Inclusion	Exclusion	Keywords
**Location**	Australia	Outside Australia	(Abstract) Australia OR “New South Wales” OR NSW OR Queensland OR QLD OR Victoria OR VIC OR Tasmania OR TAS OR Adelaide OR “Northern Territory” OR NT OR “Western Australia” OR WA
**Language**	English	Not written in English	Select for English only
**Time**	Any	None	N/A
**Population**	Aboriginal and Torres Strait Islander Children/Aboriginal Children 0–12 years old in Australia.	Non-Aboriginal Australians of any age or Aboriginal individuals older than 12 years old.	(Title) Aborigin^a^ OR “Torres Strait Islander” OR “Indigenous Australian” OR “Native Australian” AND Child^a^ OR Infant^a^ OR Infancy OR Kid^a^ OR Neonate^a^ OR Toddler^a^ OR Baby OR Babies OR Pediatric OR Paediatric
**Health condition**	Otitis media and ear disease-related pathology	Not concerned with otitis media or ear-disease-related pathology	(Title) “Otitis media” OR “Middle ear” OR “glue ear” OR “ear infection” OR Ear OR Hearing OR “bulging eardrum”
**Literature type**	Published primary research and grey literature (including qualitative, quantitative and mixed method design) included in databases indicated^a^.	Published literature not included in the databases indicated.	N/A
**Google Scholar Modified Search**			
1. Australia AND Aborigin^a^ OR “Torres Strait Islander” OR “Indigenous Australian” OR “Native Australian” AND Child^a^ OR Infant^a^ OR Infancy OR Kid^a^ OR Neonate^a^ OR Toddler^a^ OR Baby OR Babies OR Pediatric OR Paediatric AND “bulging eardrum”			
2. Australia AND Aborigin^a^ OR “Torres Strait Islander” OR “Indigenous Australian” OR “Native Australian” AND Child^a^ OR Infant^a^ OR Infancy OR Kid^a^ OR Neonate^a^ OR Toddler^a^ OR Baby OR Babies OR Pediatric OR Paediatric AND “Otitis media”			
3. Australia AND Aborigin^a^ OR “Torres Strait Islander” OR “Indigenous Australian” OR “Native Australian” AND Child^a^ OR Infant^a^ OR Infancy OR Kid^a^ OR Neonate^a^ OR Toddler^a^ OR Baby OR Babies OR Pediatric OR Paediatric AND “Middle ear”			
4. Australia AND Aborigin^a^ OR “Torres Strait Islander” OR “Indigenous Australian” OR “Native Australian” AND Child^a^ OR Infant^a^ OR Infancy OR Kid^a^ OR Neonate^a^ OR Toddler^a^ OR Baby OR Babies OR Pediatric OR Paediatric AND “ear infection”			
5. Australia AND Aborigin^a^ OR “Torres Strait Islander” OR “Indigenous Australian” OR “Native Australian” AND Child^a^ OR Infant^a^ OR Infancy OR Kid^a^ OR Neonate^a^ OR Toddler^a^ OR Baby OR Babies OR Pediatric OR Paediatric AND “glue ear”			

^a^**Databases**: PubMed, ProQuest, Scopus, Informit, Medline and Google Scholar

The literature search was conducted in April 2017. Keywords were established and agreed upon by the research team with the assistance of university library staff for the parameters: location, population and health condition. The selected databases were chosen upon consensus and the search was conducted independently by each research team member and the assisting librarian to limit bias. Boolean operators were applied in the literature search within PubMed, ProQuest, Scopus, Informit, Medline and Google Scholar. For the Google Scholar literature search, multiple searches were conducted due to search box restrictions (see Table 1). Location keywords were substituted by selecting results from Australia only and each of the OM-related terms were searched for separately. The population keywords were searched with Boolean operators consistent with other database searches and is detailed in Table 1.

### Selecting literature

The first step in selecting the literature was to exclude any duplicate papers. This was done using EndNote (electronic referencing software). Google Scholar results were limited to the first two pages, given the large number of results yielded and time constraints for conducting the literature search. An Excel spreadsheet was created to categorise the literature based on the following parameters: author, title, year, within Australia, ‘Aboriginal-related term’, ‘OM-related term’ and full text available. The literature was then systematically evaluated based on these criteria and included or excluded accordingly. Where there was any uncertainty regarding the suitability of an article, consensus on whether to include the article(s) was reached by the research team.

### Collating, Analysing and reporting results

**Fig. 1:**
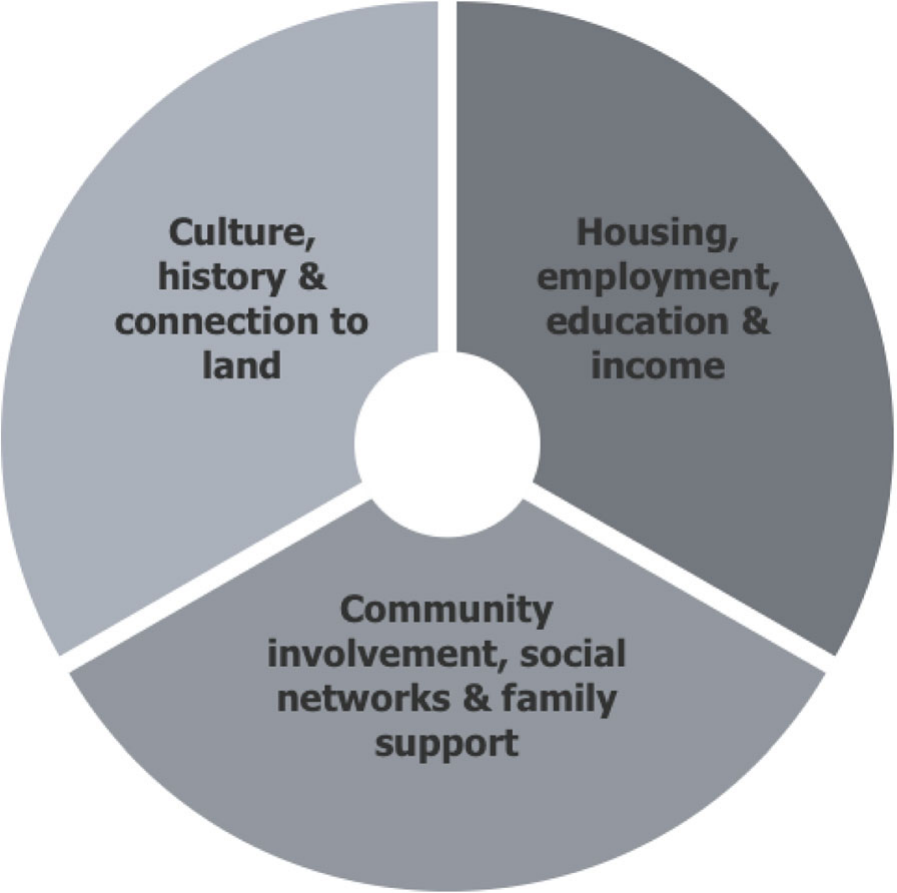
Social Determinants Framework for Aboriginal and Torres Strait Islander Health

Following selection of the included literature, two separate Excel spreadsheets were created to analyse and report the results. One spreadsheet contained the peer-reviewed literature and the other contained the grey literature. The articles were systematically analysed based on the following parameters: ‘are social determinants mentioned?’, ‘what section are social determinants mentioned?’, ‘what social determinants are mentioned?’, ‘are social determinants mentioned as drivers of OM?’, ‘are social determinants discussed in regards to interventions for OM?’, ‘what is discussed in regards to future directions?’, ‘are interventions related to social determinants or mentioned but not fundamental to the discussion or conclusion?’, and ‘are social determinants related to one of three key areas of the Aboriginal and Torres Strait Islander social determinants of health framework?’- adapted from Department of Health and Ageing (Health*Info*Net) [14], as shown in Fig. 1.

## Results

**Fig. 2:**
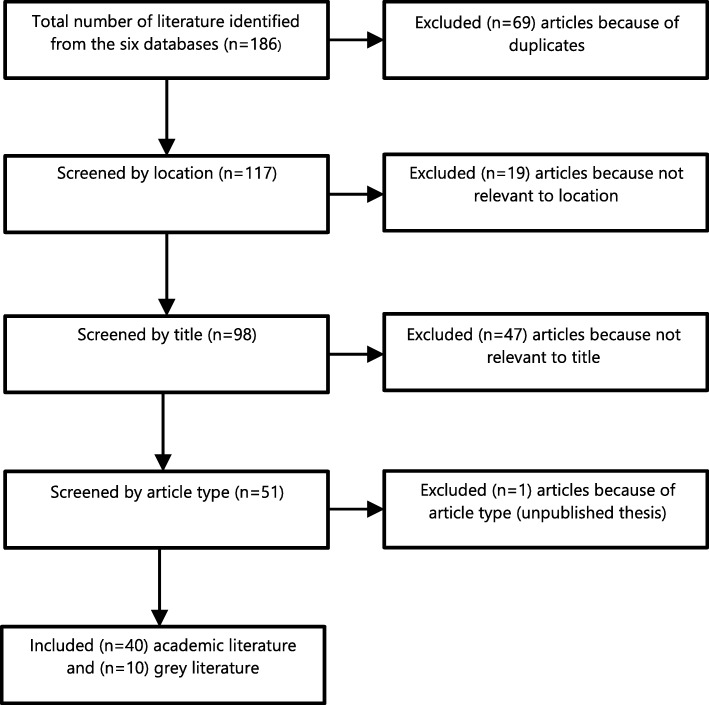
Search Strategy and Results

The literature search was conducted using six specified databases and the exclusion process is detailed in Fig. 2. The search yielded 186 results, 69 duplicates were excluded and a further 19 articles were excluded based on location of the studies. 98 articles were screened by title and article type, with 47 excluded based on irrelevance of the title and one article was excluded due to the article type (unpublished thesis). Following the screening process, 50 articles were included in the study. Of the 50 included articles, 40 were peer-reviewed and 10 were grey literature articles.

### Drivers and intervention

Following the exclusion process, the included literature was evaluated by how OM related social determinants were addressed. 34 (68%) peer-reviewed and grey articles were identified as discussing social determinants, with 17 (34%) discussing social determinants as a significant factor for driving the high rates of OM and 17 (34%) articles identifying the need to address social determinants to reduce the high rates of OM in Aboriginal children. Of the 17 articles that discuss social determinants as important for OM management, 11 articles did not discuss this in detail - these articles did not provide specific recommendation or evidence for further research and policy development. For example, Sparrow et al. [15] (p14) state “the key to improving chronic middle ear disease must be by addressing living standards and general health”. Although this type of statement is true and does acknowledge an important issue, the article does not pursue this theme further.

Further evaluation of the literature revealed that 16 (32%) articles did not mention social determinants at all, with four articles (8%) providing analysis of social determinants of OM. These four articles presented social determinants as key priority areas for future intervention and provided supported recommendations to help address social determinants linked to OM. For example, Jacoby et al. [16] (p602) state “there is a need for more input by Indigenous Australians in developing programs, increased funding and improved access to nicotine replacement therapy”. Lastly, the most significant finding was that despite the majority (68%) of the literature discussing social determinants as impacting the presence of OM in Aboriginal children, there were no studies within the literature that proposed or investigated a social determinants-focussed intervention.

### Aboriginal and Torres Strait islander social determinants of health framework

In addition to the social determinants-related scoping criteria, the literature was comprehensively assessed using the ‘Social determinants framework for Aboriginal and Torres Strait Islander health’. [14] The social determinants of health framework identifies three key areas of health for Aboriginal populations, with the literature addressing ‘housing, employment, education and income’ most frequently (32%) in relation to high rates of OM in Aboriginal children. ‘Community involvement, social networks and family support’ were discussed by few articles (16%) and even fewer mentioned ‘culture, history and connection to land’ (8%). Moreover, over 50% of the peer-reviewed articles (*n* = 22) did not address any of the three key areas of the social determinants of health framework.

### Social determinants

Housing-related social determinants were reported most frequently within the literature, with 28 (56%) reports of housing related risk factors for OM (18 specifically related to overcrowded housing). The next most frequently discussed social determinant was exposure to tobacco smoke, with 11 articles (22%) discussing this as a significant determinant for OM. Low socioeconomic status, low income and poverty (20%), access to services (18%), hygiene (16%), and education of the primary caregiver (14%) were among the most frequently mentioned determinants. Other reported determinants for OM were employment status and employment opportunities (12%), nutrition (12%), community involvement in service provision and planning (6%), and cultural and language differences (*n* = 4). Sun et al. [17] (p8) explain that improved housing for Aboriginal populations is desperately needed, as “overcrowding is the single most important and most consistent risk factor for upper respiratory tract carriage (presence of bacteria), and consequently, the development of OM in Indigenous children”. It is therefore important to note, that of the 40 peer-reviewed articles, only Jacoby et al. [18] examined overcrowded housing and its impact on OM associated bacterial carriage. Jacoby et al. [18] provide thorough analysis on aspects of overcrowding, such as the number of adults, children and rooms within a household and its impact on OM occurrence. More specifically, the greater the number of people, the greater the number of children and the fewer rooms within a house, the greater the risk of developing OM [18]. Unfortunately, this article did not identify any means to address these issues and only highlights the seriousness of the housing problems faced by many Aboriginal communities.

### Future directions

A detailed analysis was performed on what recommendations were made in the literature (i.e. review of the recommended approaches to the management of OM in Aboriginal children). 31 (62%) of the peer-reviewed and grey articles did not discuss social determinants in future directions at all. 23 articles (46%) primarily recommended further research into antibiotic treatment and vaccine development, and the need for greater understanding of OM associated bacterial carriage. Five (10%) articles presented detailed recommendations for future research and policy development intended to address social determinants to reduce the high rates of OM in Aboriginal children.

## Discussion

OM is one of the leading causes of preventable disease amongst Aboriginal children, and has been determined by The World Health Organisation to be a serious public health issue requiring urgent attention [1–3, 11]. OM primarily occurs during developmental years and can drastically impact upon speech and language development, which is likely to influence educational outcomes and prospective employability- precursors to potentially life-long socioeconomic disadvantage and poverty [5].

This study identifies how social determinants are addressed within grey and peer-reviewed literature, and summarises the primary determinants reported to be associated with OM and management recommendations within the literature. This study highlights gaps between factors reported to be associated with OM and recommended interventions within the literature. Given the significance of this gap, further research aimed at understanding social determinants associated with OM and identifying more effective management of the social determinants of OM within Aboriginal children is warranted. Furthermore, the inter-related nature of the social determinants of health is emphasised throughout this paper and helps to underline the challenge that an exclusively biomedical model poses in addressing specific aetiology [19].(p73–74)

Notably, a shift in approaches to manage OM is desperately needed, in conjunction with further research to better understand the relationship between the social determinants of health and risk of OM in Aboriginal populations. This review demonstrates that there is an imbalanced research focus towards biomedical approaches in contrast to improving our understanding about how to address key social determinants contributing to high rates of OM in Aboriginal children. Using the social determinants of health framework, this review has identified significant shortcomings within the literature and the current public health management of OM in Aboriginal children. The social determinants of health framework used within this study identifies three key areas of Aboriginal health that are largely neglected by the available grey and peer-reviewed literature in relation to OM management. Although the literature mentions various social determinants that are consistent with the framework (e.g. housing, education, employment, community engagement, culture and history), none of the included articles evaluated these key areas of Aboriginal health with the objective to establish effective social, environmental, political or cultural-focussed interventions for OM. Further, the key social determinants of OM can be argued to stem from the persistent social, economic and cultural discrimination experienced by Aboriginal populations. Through evaluation using the social determinants of health framework, this review highlights the need to preserve Aboriginal culture, strengthen Aboriginal self-determination, respect and support Aboriginal connection to land, empower Aboriginal communities, improve education and employment opportunities for Aboriginal people, and address poor housing conditions and overcrowding within Aboriginal communities. Importantly, one of the most significant and achievable goals should be to ensure the adoption of co-creation and a decolonised approach to ear health research, and health research more broadly, in Aboriginal populations. Aboriginal self-determination and services that are embedded within community are key to improving the management of OM within Aboriginal populations [20]. Such an approach is needed to help ensure success of public health programmes and services aimed at reducing the risk of OM in early life, and consequently helping to eliminate the cycle of disadvantage that contributes to social determinants driving ill-health across the life-course. Measurement of such targets should be done through formal and informal consultation with community at each step of the research process. There is growing acknowledgement within the literature that the current empirical research paradigm should adopt co-creation and qualitative research methods, in conjunction with quantitative methodology, to ensure successful research and research translation within Aboriginal communities [20]. Furthermore, recognising Aboriginal people as experts of their communities is vital to ensure successful planning, development, implementation and evaluation of health research and health approaches within Aboriginal contexts.

The most evident theme arising from this review was the importance of the home environment, with housing-related determinants reported almost three times more than the next most frequently reported risk factor. Despite acknowledgement of the association between housing and the prevalence of OM in Aboriginal children, there were no intervention studies within the reviewed literature that investigated how to effectively address the issue of housing in Aboriginal populations. Exposure to cigarette smoke and poor hygiene were not directly acknowledged as relating to housing within this review. However, these risk factors are likely to be influenced to some degree by the home environment, given the relatively high rates of smoking within the home in Aboriginal populations [15, 16]. It is therefore evident, that addressing the home environment is fundamental to adequately manage OM in Aboriginal populations. Moreover, further research into housing as a determinant of OM and as a means for intervention is desperately needed, given the lack of information available to adequately deal with this area of Aboriginal health. Addressing housing issues in Aboriginal communities is a complex issue, particularly when considering the importance of connection to land in contrast with the importance of the physical structure itself. It can be said that the efforts of government housing programmes have been heavily focussed on the logistics. For example, funding and physical infrastructure, with little acknowledgement of the need to develop culturally appropriate housing policies and pathways [21]. (p207) Carson et al. [21] (p219) stress the lack of intervention studies that link housing to Aboriginal health outcomes and the ability to develop policy is limited as a result. The lack of intervention studies is also highlighted by this review, as no intervention studies looking at social determinants and Aboriginal health outcomes were identified within the literature. Intervention studies are crucial for policy development and although remoteness, and political and social barriers exist for improving housing and infrastructure in Aboriginal communities [21], a shift in focus towards more culturally appropriate housing policy and provision is urgently needed.

Exposure to tobacco smoke is consistently reported as a key contributing factor for Aboriginal children developing OM. Aboriginal children who are exposed to tobacco smoke in the home and who do not attend day-care have been suggested to be at greatest risk of developing OM [18]. This is not to say that home-care by parents and family is problematic. However, given the relatively high rates of smoking within the home environment [18], it is an important issue for consideration. Jacoby et al. [18] suggest that children who are exposed to tobacco smoke in the home who also attend day-care may be at lower risk of developing OM, presumably because the time spent at day-care means less time exposed to tobacco smoke in the home. However, day-care attendance has previously been associated with a greater risk of OM, and further research may help to explain this relationship. Moreover, this inconsistent research helps to highlight the evident gaps within the literature resulting from the long-standing narrow lens of the biomedical focus of the existing research. Furthermore, this supports calls for further investigation into the relationship of the social determinants of health and environmental factors with OM risk in Aboriginal children.

Education and employment of the primary caregiver is cited frequently as an important determinant for Aboriginal children developing OM. However, no paper within the reviewed literature discussed this any further than listing it as a significant contributing factor. It is important to highlight that low-level education and lack of employment opportunities consign many Aboriginal people to levels of poverty [22]. (p108) Furthermore, education that excludes culture and native language has been demonstrated to adversely impact individuals by disempowering Aboriginal communities and harming the cultural identity of these communities [21]. Moreover, hearing loss associated with OM is likely to further disengage children within the classroom, and this is compounded by lack of engagement due to hearing loss being misconstrued as misbehaviour. It is therefore clear, that Aboriginal children face significant barriers within the classroom and highlights the need for culturally appropriate schooling, accompanied by approaches to reduce rates of OM and hearing loss. Notably, there were no papers identified within this review that comprehensively evaluated the impact of OM across the life-course, including the impact of OM on speech, language and early childhood development, which may impact educational outcomes and long-term social and emotional wellbeing.

Aboriginal community involvement is an area that requires greater emphasis and encouragement from public health promoters, policy makers and service providers. Programmes such as the ‘Healthy Ears, Happy Kids’, [9] ‘Aboriginal Otitis Media Project’ [23], ‘Hearing, Ear Health and Language Services’ (‘HEALS’) [24] and ‘Deadly Kids, Deadly Futures’ [25] help to draw attention from government and non-government organisations towards the seriousness of the burden of OM in Aboriginal communities. ‘HEALS’ and ‘Deadly Kids, Deadly Futures’ have helped to demonstrate priority areas for the public health management of OM in Aboriginal communities, in addition to recommendations about key research considerations when working with Aboriginal communities. Priorities include working towards improved coordination, access and delivery of services, enhancing capacity building within communities, and Aboriginal control of research activities and translation [24, 25]. Furthermore, these programmes have helped to educate and empower Aboriginal communities and health workers to manage OM more effectively in a culturally safe way [9, 23, 24]. Given the historical marginalisation, neglect and subjugation of Aboriginal populations, empowering Aboriginal communities to manage health services, develop and implement research, and provide recommendations is essential to overcome issues of mistrust, and consequently, improve cultural access to essential services. Importantly, ‘Deadly Kids, Deadly Futures’, which was not identified by the systematic literature search, provides a ‘social determinants model of ear and hearing health’ that highlights relevant social determinants of ear health for Aboriginal children [25]. This model may be useful to guide future research, policy development and the development of services. However, research focussing on how to best target these social determinants is lacking. Therefore, further work is needed to advance these programmes and identify how to effectively target the underlying social determinants of OM in Aboriginal children.

Despite the lack of research about how to effectively target the social determinants of OM, there is a growing body of research regarding diversifying health approaches to better address social determinants of health more broadly. The term ‘Integrated models of care’ has emerged within the literature, which describes the integration of biomedical services with non-medical community services (e.g. housing, employment and food insecurity services) to provide a more comprehensive approach to target underlying risk factors for ill-health [26]. Using a similar approach, it is recommended that tools to screen for social determinants associated with OM are developed. This will assist health workers to identify and target important social, environmental and cultural risk factors associated with OM [27, 28]. Information obtained through this type of screening may provide health workers with relevant information to refer at-risk children to community services, in conjunction with traditional medical management, to help alleviate factors placing a child at heightened risk. This process has been referred to as ‘social prescribing’ and aims to broaden the often-narrow focus of biomedical intervention alone [28]. Therefore, it is recommended that future research looks at ‘integrated models of care’ and ‘social prescribing’, and how they can be incorporated into primary care management of OM and ear disease. Additionally, service coordination is key for successful navigation of healthcare systems and referral pathways, which are often complex. By integrating a wider variety of services in the primary care of OM, such as housing or employment services, the need for coordination is particularly important to support the implementation of such models [24, 28].

While this review presented a comprehensive analysis of both peer-reviewed and grey literature, this study excluded unpublished masters and doctoral theses. Despite this, findings by Vickers and Smith [29] following review of the Cochrane Library, found only one of 878 systematic reviews included data from theses that could have significantly altered the conclusions of the 878 reviews. Moreover, there is limited benefit of including theses in systematic reviews, as they rarely influence the conclusions, and retrieving and analysing unpublished dissertations involves considerable time and effort [29]. The timeframe of this project also limited the number of selected databases and consequently the number of papers that were included within the study. However, 50 articles still provides comprehensive scope of the literature to enable thorough analysis, detailed explanation and well supported recommendations. Using Google Scholar presented limitations in search function, as search box options within the database meant that a modified search was needed to fulfil the specified search strategy and to remain consistent with searches performed on the other selected databases.

## Conclusion

There is overwhelming consensus within the reviewed literature that Aboriginal children experience disproportionately high rates of OM when compared to non-Aboriginal children. The high rates of OM are linked to poor housing conditions, overcrowded housing, exposure to tobacco smoke, education, and overall social and economic disadvantage. Furthermore, there is disparity between reported risk factors of OM and current interventions aimed at reducing the burden of OM in Aboriginal populations. Current interventions are primary focussed on biomedical approaches such as investigating vaccines and antibiotics. Although vaccines and antibiotics are essential to the provision of high-quality clinical care for OM, a broader public health lens is required to address the underlying social factors reported to be driving the gap in OM rates between Aboriginal and non-Aboriginal children. It is important to mention that the Aboriginal understanding of health includes “body, mind, spirit, land, environment, custom, socioeconomic status, family and community” [10]. (p417) This understanding of health significantly differs from mainstream models of health, which typically involves the pursuit to merely limit ill-health within individuals without considering the context of their lives [10]. Therefore, policy and services founded upon this restricted understanding of health is likely to be restrictive in its ability to address the much more holistic Aboriginal understanding of health, which includes how people live, work and interact with their environment, and the importance of community for the individual. In accordance with this notion, engaging communities in research design and implementation is fundamental to shift the current research paradigm. Understanding the context of Aboriginal lives is key for successful research and meaningful translation of research. Further research into how social determinants contribute to OM and what interventions may be beneficial to address OM associated social determinants in Aboriginal children is needed. Intervention studies to evaluate the benefit of culturally suitable, accessible and safe housing on rates of OM in Aboriginal communities is vital. Lastly, development of an Aboriginal ear health framework is recommended. Development of a comprehensive ear health framework requires further research, although should include information about social determinants of health screening, social prescribing, and coordinating the complex network of health and community services that may help to address underlying social determinants of OM.

**Table:** 

**Recommendations and Future Directions**	
Research evaluating the association between social determinants of health and risk of OM in Aboriginal children	
Research evaluating the consequences of OM across the life course	
Development of a co-created social determinants of ear health framework including: • The development of social determinants of health screening tools • The development of a social prescribing model • The development of a service navigation and coordination model	
Evaluate if approaches targeting the social determinants of ear health reduce rates of OM in Aboriginal children	

## Data Availability

Data are available through the corresponding author.

## Abbreviations

OM: Otitis Media
Aboriginal and Torres Strait Islander: Aboriginal
